# Confusion remains an important issue in public goods game experiments

**DOI:** 10.1073/pnas.2411093121

**Published:** 2024-07-25

**Authors:** Eirik Strømland, Lina Koppel, Magnus Johannesson, Gustav Tinghög

**Affiliations:** ^a^HVL Business School, Western Norway University of Applied Sciences, 5020 Bergen, Norway; ^b^Department of Management and Engineering, Linköping University, 58183 Linköping, Sweden; ^c^Department of Economics, Stockholm School of Economics, 11383 Stockholm, Sweden

The article “Confusion cannot explain cooperative behavior in public goods games” by Wang et al. (1) is based on two experiments that modify and replicate Burton-Chellew, El Mouden & West (BEW) (2). Wang et al. implement a pregame quiz about the game’s incentives sometimes used in the literature (3), but in contrast to BEW, subjects cannot proceed until they have answered the questions correctly. After this quiz, Wang et al. measure comprehension and report that only 2.5% (Study 1) to 4% (Study 2) misunderstand the game. They argue that their findings undermine BEW’s conclusion, suggesting that confusion is not an important factor in explaining cooperative behavior.

This interpretation is, in our view, misleading. In study 2, Wang et al. find that 74% of subjects answer BEW’s comprehension question incorrectly even after correctly answering the pregame quiz. Further consistent with confusion, Wang et al. find a pattern of increasing contributions even in the Computer condition. To reconcile this with their interpretation, Wang et al. argue that BEW’s question is “not directly relevant to the misunderstanding of the game” (p. 5). However, someone who understands the public goods game should answer both questions correctly. The two questions are

Wang et al.: “*In a one-shot game, given that the amount contributed to the project by the other group members of your group is 30 MU, if you want to maximize your own benefit, how much should you contribute to the project (of course, your actual contribution may be different)?*”

BEW: “*In the game, if a player wants to maximize his or her earnings in any one particular round, does the amount they should contribute depend on what the other people in their group contribute?*”

Importantly, BEW’s question captures an additional source of confusion not captured by Wang et al.’s question: Subjects may incorrectly believe that their choice can influence the choice of others within the same round. For instance, a subject may understand the one-shot nature of the game but mistakenly believe that she is playing a sequential PGG, where it is true that the amount the first mover contributes can influence the contribution of the other group members.

Wang et al. report that there is no significant correlation between the distribution of behavior types and confusion on the BEW question for the Wang et al. data, but the statistical power to detect this with their sample size (N = 72) is very low. A study by Fosgaard et al. (4) asks a similar comprehension question as Wang et al. and report that about 40% of participants answer incorrectly (although they had as in Wang et al. passed a number of quiz questions prior to the game). Their sample is very large (N = 2,042). They also report that the distribution of behavior types is substantively affected by subjects’ confusion status. Their findings raise questions concerning the generalizability of the Wang et al. results. In summary, confusion remains an important issue, even when explicit attempts have been made to eliminate it.
